# Selenoprotein P Concentrations in the Cerebrospinal Fluid and Serum of Individuals Affected by Amyotrophic Lateral Sclerosis, Mild Cognitive Impairment and Alzheimer’s Dementia

**DOI:** 10.3390/ijms23179865

**Published:** 2022-08-30

**Authors:** Teresa Urbano, Marco Vinceti, Jessica Mandrioli, Annalisa Chiari, Tommaso Filippini, Roberta Bedin, Manuela Tondelli, Cecilia Simonini, Giovanna Zamboni, Misaki Shimizu, Yoshiro Saito

**Affiliations:** 1 CREAGEN—Environmental, Genetic and Nutritional Epidemiology Research Center, Department of Biomedical, Metabolic and Neural Sciences, University of Modena and Reggio Emilia, 287 Via Campi, 41125 Modena, Italy; 2 Center for Neurosciences and Neurotechnology, Department of Biomedical, Metabolic, and Neural Sciences, University of Modena and Reggio Emilia, 287 Via Campi, 41125 Modena, Italy; 3 Department of Epidemiology, Boston University School of Public Health, 715 Albany Street, Boston, MA 02118, USA; 4 Neurology Unit, Azienda Ospedaliero Universitaria di Modena, 71 Via del Pozzo, 41121 Modena, Italy; 5 School of Public Health, University of California Berkeley, 1995 University Avenue, Berkeley, CA 94704, USA; 6 Laboratory of Molecular Biology and Metabolism, Graduate School of Pharmaceutical Sciences, Tohoku University, Sendai 980-8578, Japan

**Keywords:** selenium, selenoprotein P, mild cognitive impairment, dementia, amyotrophic lateral sclerosis

## Abstract

Selenoprotein P, a selenium-transporter protein, has been hypothesized to play a role in the etiology of neurodegenerative diseases such as amyotrophic lateral sclerosis (ALS) and Alzheimer’s dementia (AD). However, data in humans are scarce and largely confined to autoptic samples. In this case–control study, we determined selenoprotein P concentrations in both the cerebrospinal fluid (CSF) and the serum of 50 individuals diagnosed with ALS, 30 with AD, 54 with mild cognitive impairment (MCI) and of 30 controls, using sandwich enzyme-linked immunosorbent assay (ELISA) methods. We found a positive and generally linear association between CSF and serum selenoprotein P concentrations in all groups. CSF selenoprotein P and biomarkers of neurodegeneration were positively associated in AD, while for MCI, we found an inverted-U-shaped relation. CSF selenoprotein P concentrations were higher in AD and MCI than in ALS and controls, while in serum, the highest concentrations were found in MCI and ALS. Logistic and cubic spline regression analyses showed an inverse association between CSF selenoprotein P levels and ALS risk, and a positive association for AD risk, while an inverted-U-shaped relation with MCI risk emerged. Conversely, serum selenoprotein P concentrations were positively associated with risk of all conditions but only in their lower range. Overall, these findings indicate some abnormalities of selenoprotein P concentrations in both the central nervous system and blood associated with ALS and neurocognitive disorders, though in different directions. These alterations may reflect either phenomena of etiologic relevance or disease-induced alterations of nutritional and metabolic status.

## 1. Introduction

Amyotrophic lateral sclerosis (ALS) is an extremely severe neurodegenerative disease characterized by the progressive degeneration of motor neurons in both the brain and the spinal cord [1]. ALS cases are regarded as being familial and/or due to inherited gene mutations in 10–15% of cases, while the remaining 85–90% of cases are sporadic, with far less evidence of a role of genetic factors and the likely involvement of one or more environmental and lifestyle determinants [1]. Alzheimer’s dementia (AD) is a much more common neurodegenerative disease, clinically characterized by global cognitive impairment significantly affecting everyday functioning. The underlying neuropathology, named Alzheimer’s disease, is characterized by amyloid plaque deposition and neurofibrillary tau tangles. Both disease incidence and prevalence increase with age and are predicted to rise in the next years, mainly due to life expectancy increase [2,3]. As with other dementia forms, AD is preceded by a prodromal condition named mild cognitive impairment (MCI), characterized by cognitive decline without a significant impact on functional independence. MCI sometimes, but not always, progresses to dementia [4,5].

Among the environmental factors hypothesized to be involved in the etiology of neurodegenerative disease, possibly by interacting with genetic factors, are heavy metals and metalloids, pesticides and other chemicals [6,7,8,9,10,11,12]. These potential risk factors include selenium, a metalloid with both nutritional and toxic properties depending on the dose and compound considered [13,14]. This trace element has long attracted researchers from all over the world, who have thoroughly debated its human health effects [14,15]. Although few epidemiologic studies have investigated the effects of too low and too high selenium exposure on the human nervous system, current evidence suggests that, while the absence of selenoproteins may have highly deleterious effects on the central nervous system, neurotoxic effects may derive from acute and chronic selenium overexposure, even at unexpectedly low levels [16,17]. For instance, studies carried out in seleniferous areas in China indicated that peripheral anesthesia, seizures and disturbances such as paralysis, hyperreflexia and polyneuritis can follow chronic selenium intoxication [14,18], while short-term severe overexposure may lead to acute neurotoxicity [19,20]. On the other hand, selenium [21,22,23] and selenium-containing nanoparticles [24,25,26,27] have been suggested by laboratory and animal studies to play a beneficial role in the etiology, prevention and the therapy of neurological diseases, though uncertainties still exist about the overall effects and safety of increasing brain selenium exposure [28,29].

In addition to the uncertainties concerning the actual effects of overall selenium exposure on the nervous system, little is known about the single selenium compounds directly involved in the risk of neurological disorders, as well as the selenoproteins whose deficit and excess may be specifically implicated in such a relationship, including selenoprotein deficiencies due to gene mutations [21,22,23]. Very limited evidence is also available about the best indicators to assess selenium status inside the central nervous system. From this perspective, there has recently been a spate of interest in the selenium-transporter enzyme selenoprotein P. This has been suggested to have antioxidant properties and to share the fate of the trace element itself. In other words, selenoprotein P has been considered as potentially beneficial or adverse also depending on its levels and the specific endpoints considered [30,31,32,33]. Selenoprotein P is encoded by *SELENOP* and mainly produced in the liver [34]. Moreover, it contains multiple selenocysteine residues, which makes it unique among selenoproteins [35]. In recent years, suggestive evidence has been provided supporting the notion of an adverse effect of selenoprotein P on metabolic disease risk, and particularly in the induction of type 2 diabetes and other disorders such as vascular disease [36,37,38,39,40]. On the other hand, little evidence is available about its relationship with neurological diseases in humans, despite the considerable interest in this topic [21,23,41,42,43,44,45]. In vitro, selenoprotein P expression and availability has also been reported in several brain cell types, including neurons and ependymal cells [32].

In this study, we aimed at assessing for the first time CSF selenoprotein P content in vivo in patients with newly diagnosed ALS, AD and MCI as well as in controls and at characterizing the possible involvement of CSF and blood selenoprotein P concentrations in the etiology and the progression of these neurodegenerative diseases.

## 2. Results

Based on CSF sample availability, we eventually recruited 164 subjects (87 men and 77 women). Of these, 30 were diagnosed with AD and 50 with ALS; 54 individuals with MCI and 30 controls were also included. For all ALS patients and for controls, a serum sample was also available, while a serum sample was not available for four AD patients and seven individuals with MCI. In controls, CSF selenoprotein P levels were 22.1 ng/mL (interquartile range (IQR) 14.9–29.9 ng/mL), with higher values in males (28.7 ng/mL) compared to females (19.1 ng/mL) and in participants aged ≥65 years (29.5 vs. 20.1 ng/mL). Selenoprotein P levels in the serum amounted to 5615.9 ng/mL (IQR 4683.6–6235.7 ng/mL), with higher values in females (5694.0 ng/mL) than in males (5310.8 ng/mL). When considering the overall population, similarly, males exhibited higher CSF and serum levels of selenoprotein P compared to females (Table S1).

Selenoprotein P median and IQR concentrations in serum and CSF according to diagnosis are reported in Table 1, alongside selenoprotein-P-bound selenium levels and neurodegeneration (t-tau, p-tau181) and amyloidosis (Aβ1-42) biomarkers (available only for MCI and AD participants).

The highest CSF selenoprotein P levels were found in the individuals with MCI and AD and the lowest in patients with ALS, while for serum, the highest levels emerged in the MCI subgroup and the lowest in controls. Individuals with MCI exhibited slightly higher selenoprotein-P-bound selenium levels compared to AD patients. Concerning neurodegeneration and amyloidosis biomarkers, individuals with MCI had higher average Aβ1-42 levels and lower t-tau and p-tau181 concentrations, compared with AD patients.

**Figure 1:**
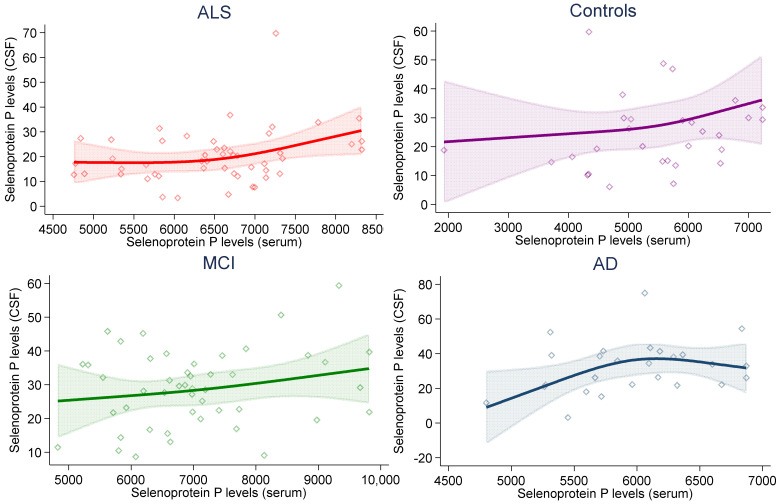
Association between serum and cerebrospinal fluid (CSF) selenoprotein P levels (ng/mL) in study subgroups in multivariable (age- and sex-adjusted) spline regression analysis, with 95% confidence limits.

Figure 1 reports the results of spline regression analysis to assess the associations between CSF and serum selenoprotein P levels, after adjusting for age and sex. Such levels were positively and nearly linearly correlated in each study subgroup, except for patients with AD, for whom, an inverted U-shaped association emerged, with a positive association until 6000 ng/mL of serum levels, then followed by a slight decrease. In multivariable linear regression analysis, the β coefficient was 0.003 (95% CI 0, 0.007), 0.002 (−0.001, 0.004), 0.003 (−0.004, 0.011) and 0.003 (−0.001, 0.007) in the ALS, MCI, AD and control group, respectively.

**Figure 2:**
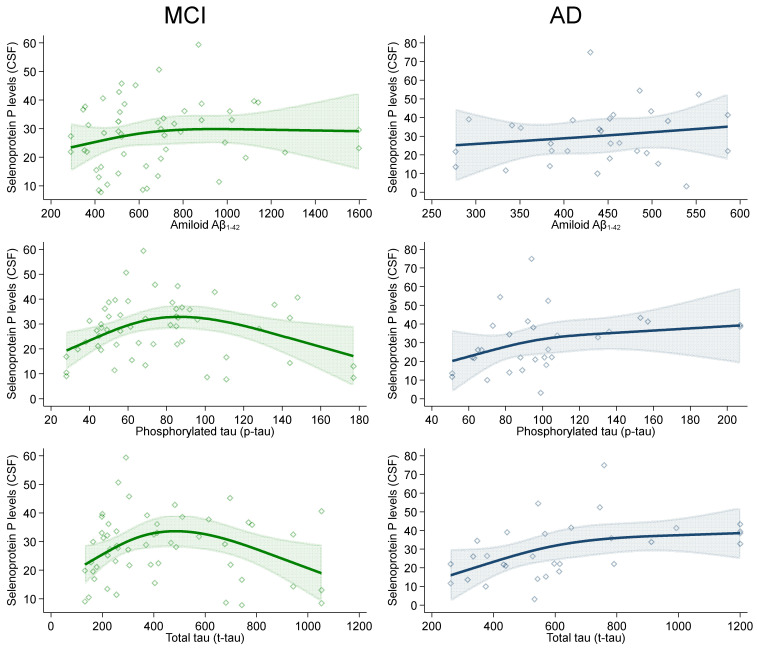
Association between selenoprotein P levels (ng/mL) and neurodegeneration biomarkers (ng/L) in cerebrospinal fluid (CSF) in individuals with Alzheimer’s dementia (AD) and mild cognitive impairment (MCI) in spline regression analysis adjusted for age, sex and education, with 95% confidence limits.

In the spline regression analyses between CSF neurodegeneration and amyloidosis biomarkers and CSF selenoprotein P levels, Aβ1-42 levels slightly and positively correlated with selenoprotein P in the AD group. This association tended to also be positive in MCI individuals until 800 ng/L of selenoprotein P concentrations, before the curve flattened (Figure 2).

Patterns for p-tau181 and t-tau levels in relation with selenoprotein P were substantially similar in the AD group, while in the MCI one, at high levels of both neurodegeneration biomarkers, we found an inverted-U-shaped pattern with turning points at around 80 and 500 ng/L of p-tau181 and t-tau, respectively.

**Figure 3:**
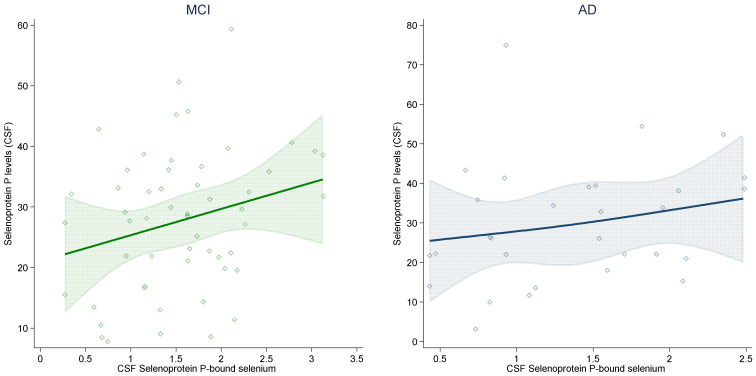
Association between selenoprotein P levels and selenoprotein-P-bound selenium in cerebrospinal fluid (CSF) (ng/mL) in individuals with mild cognitive impairment (MCI) and Alzheimer’s dementia (AD) in spline regression analysis adjusted for age, sex and education, with 95% confidence limits.

With reference to the association between selenoprotein-P-bound selenium and selenoprotein P concentrations in CSF (Figure 3), as shown by spline regression analysis, after adjustment for potential confounders, the relation was positive, strong, almost linear and statistically imprecise in both groups (AD and MCI) for which these paired analytical determinations were available. In adjusted linear regression analysis, the β coefficient was 4.28 (95% C.I. −0.26, 8.82) in MCI and 5.33 (95% C.I. −3.49, 14.15) in AD.

In the logistic regression analysis (Table 2), we found a negative association between a 10-unit increase in CSF selenoprotein P concentrations and ALS risk in both crude and adjusted models, while there was little association with serum selenoprotein P.

**Figure 4:**
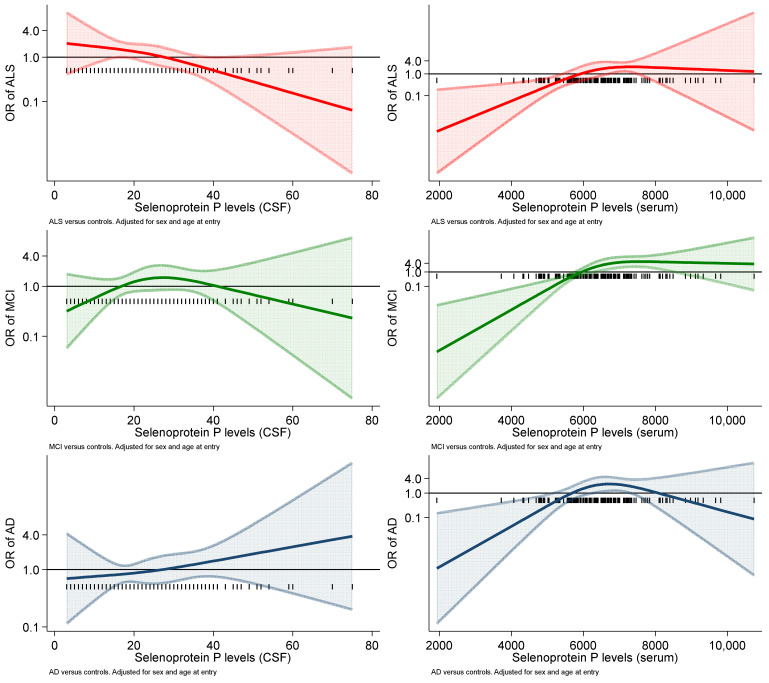
Association of selenoprotein P levels in cerebrospinal fluid (CSF) and in serum (ng/mL) with the odds ratio (OR) of Alzheimer’s dementia (AD), mild cognitive impairment (MCI) and amyotrophic lateral sclerosis (ALS) in spline regression analysis, with 95% confidence limits. The reference line at 1.0 with black spikes indicates the levels of selenoprotein P in the study participants.

Conversely, the association between CSF selenoprotein P levels and both AD and MCI risk was positive, while the corresponding risk estimates for serum selenoprotein P levels were much attenuated and close to unity. In the spline regression analysis (Figure 4), the relation between CSF selenoprotein P and risk was roughly linear over the entire range of exposure tested for both ALS and AD, although in the opposite direction (negative for the former and positive for the latter), while the pattern for MCI risk was inconsistent, showing an inverted-U-shaped pattern.

The spline analyses for serum selenoprotein P concentrations yielded considerably different results, with a positive association in the lowest range and no or an inverse association at higher concentrations for the ALS-MCI and AD groups, respectively.

## 3. Discussion

This study about selenoprotein P concentrations in individuals affected by neurodegenerative disease is apparently the first ever to have been carried out in vivo and using a biomarker within the central nervous system, in addition to the serum levels of this protein. We found that relatively high CSF selenoprotein P concentrations characterized both prevalent AD and the milder degree of cognitive impairment represented by MCI, while in spline and logistic regression analysis, there was a stronger and monotonic association with AD risk compared with the inconsistent relation with MCI risk. This suggests that the occurrence of cognitive impairment and particularly of dementia is associated with alterations of selenoprotein P concentrations, which could be either a consequence of or a risk factor for these conditions. The considerably more inconsistent and variable pattern for MCI could be due to the fact that this condition is extremely heterogeneous, since it includes individuals not only with Alzheimer’s pathology who will likely progress to AD but also with other diseases causing cognitive impairment, and even subjects who may subsequently revert to “normal” cognitive status [46].

Conversely, ALS was associated with considerably lower CSF selenoprotein P levels compared to controls and clearly even more to AD and MCI. This suggests either an effect of prevalent disease in selenoprotein P expression or a phenomenon of etiologic interest, possibly associated with an inadequate supply of selenium to the brain for selenoprotein synthesis, and therefore, a decreased capacity to counteract the oxidative stress anticipating and characterizing this disease [47,48].

However, interpreting associations between selenoprotein P concentrations in patients and disease risk as causal could be misleading. This is so both in the central nervous system (with reference to CSF concentrations) and in blood (as indicated by serum levels) for patients affected by a major neurodegenerative disease such as AD and ALS, due to an issue of reverse causation linked to altered nutritional status and metabolism. Higher CSF and serum selenoprotein P concentrations in patients could be due, for instance, to a higher degree of oxidative stress compared with controls, with a higher need for selenium availability for antioxidant selenoprotein synthesis and, therefore, of selenoprotein P as the main selenium transporter. However, since in ALS patients, such an increase was found for blood selenoprotein P concentrations but not for its CSF concentrations, mechanisms influencing selenoprotein P status even within the same patients could considerably differ, as well as the relation of this protein with disease risk.

Contrary to the aforementioned findings for CSF selenoprotein P content, the relation between selenoprotein P concentrations in the serum and the risk of neurodegenerative disease systematically suggested a positive association at the lower range of protein levels, and no association (or for AD risk even an inverse relation) above an approximate threshold of 6000 ng/mL. These findings are difficult to interpret with reference to the etiology of ALS, AD and MCI, limiting the utility of determining circulating selenoprotein P levels for the diagnosis and follow up of neurodegenerative disorders. They also suggest an uncoupling of the blood and CSF selenoprotein P concentrations, possibly due to altered selenium availability and metabolism and disturbances of the brain–blood barrier. Therefore, despite some partial correlation between blood and CSF selenoprotein P content [49,50], only the latter appear to be linearly associated with the risk of the two major neurodegenerative diseases investigated in this study, ALS and AD, though in an entirely different direction.

The positive association between selenoprotein P levels and selenoprotein-P-bound selenium in the CSF was expected, comparable in AD and MCI participants and substantially linear. However, it also showed some statistical imprecision and variability around its basic, monotonic trend. This could reflect an uneven distribution of selenoprotein P selenium contents across participants, due to a degree of variability in the number of selenium atoms incorporated in the protein [51,52,53,54,55,56]. As far as we know, this is the first in vivo assessment of the association between these two closely related biomarkers of selenium exposure in humans, in the CSF or other compartments.

We found different patterns of association between CSF selenoprotein P levels and biomarkers of neurodegeneration, which defy simplistic interpretation. The positive associations detected in AD patients could reflect a reactive phenomenon linked to an increased need for selenium and synthesis of this key transporter of the trace element, to generate a compensatory response to counteract oxidative stress [41,57]. Alternatively, it could reflect a detrimental effect of selenoprotein P in the neurodegenerative process. This, however, is not supported by the positive relation with CSF amyloid content in AD patients, and it is difficult to assess given the potential for reverse causation of the cross-sectional study design.

The paired analysis of selenoprotein P concentrations in both CSF and serum showed some positive association in controls and in subjects with MCI and ALS but not in patients with AD, possibly due to metabolic reasons or alterations of the blood–brain barrier. However, even in the former groups, the association between CSF and serum selenoprotein P concentration was neither strong nor statistically precise, as largely expected on the basis of the little evidence available [49,50]. Overall, these findings indicate that even if central nervous system selenoprotein P content can be roughly estimated by assessing a much more easily available indicator (its serum concentration) the inferences about disease risk that can be made using the two indicators clearly differ.

In conclusion, results of our case–control study indicate that concentrations of selenoprotein P in the CSF are decreased in ALS and increased in AD and MCI, thus suggesting alterations of the overall selenium availability in the brain and/or deleterious effects of abnormal selenoprotein P concentrations. Our findings also show that CSF selenoprotein P concentrations tend to linearly correlate with biomarkers of neurodegeneration in AD but not in MCI and have a considerably more linear, monotonic pattern of association with both ALS and AD risk as compared with the blood concentrations of this protein. To reliably assess the causes and the effects of abnormalities in selenoprotein P concentrations in patients with neurodegenerative diseases, studies with a prospective design are clearly needed.

## 4. Materials and Methods

### 4.1. Study Population

For this study, we selected all subjects diagnosed with AD and ALS as well as individuals with MCI at the Neurology Clinic of Policlinico University Hospital in Modena, Northern Italy. The study period spanned 2007–2020. Diagnosis of the aforementioned conditions was performed used clinical criteria reported in detail elsewhere [9,58]. Briefly, for ALS, we included only patients with the clinically definite and probable form of the disease according to El Escorial revised criteria [59]. For MCI, we included either the amnestic form (single domain or multiple domain) or the non-amnestic form of non-vascular origin according to Peterson’s criteria revisions proposed by Winblad et al. [60,61]. For AD, we included patients meeting the criteria for probable Alzheimer disease of the National Institute of Neurological and Communicative Disorders and Stroke (NINCDS) and the Alzheimer’s Disease and Related Disorders Association (ADRDA) [62,63]. To be selected for the present study, patients had to have at least 200 µL of CSF and, whenever possible, of serum available in 2021 at the “Neurobiobanca di Modena”’ of the University of Modena and Reggio Emilia. In addition, we included subjects admitted to the clinic who underwent neurological assessment including CSF and serum sampling but were later diagnosed as not affected by neurological disease, provided that 200 µL of CSF and serum was available in mid-2021. All individuals provided written informed consent for the use of CSF and serum samples for scientific research purposes in addition to diagnostic procedures, while the Modena Ethics Committee also approved the investigation of selenium status (approval no. 84/2015 for MCI-AD and 92/2012 for ALS). From the medical records for each participant, we extracted details of medical history, along with sociodemographic characteristics such as sex, place and date of birth, residence and educational attainment.

### 4.2. Sample Collection

Each sample, CSF or serum, was collected in the morning in fasting subjects who underwent lumbar puncture and venipuncture according to standard clinical and operating procedures. Each sample was received by the adjacent Neurology Laboratory within 30 min from collection and was centrifuged at 2500× *g* for 10 min at controlled room temperature. After centrifugation, samples were aliquoted into polypropylene sterile tubes and stored at −80 °C awaiting testing. CSF amyloid beta 1-42 (Aβ1-42), phosphorylated (p-tau181) and total tau protein (t-tau) were determined with the enzyme-linked immunosorbent assay (ELISA) method (INNOTEST^®^ β-AMYLOID(1-42), INNOTEST^®^ hTAU-Ag and INNOTEST^®^ PHOSPHO-TAU(181P), Innogenetics, Ghent, Belgium) following the manufacturers’ instructions. For the purpose of selenoprotein P determination, the remaining aliquots of each sample were then transported deep frozen in dry ice by air courier to the Molecular Biology and Metabolism Laboratory of Tohoku University (Sendai, Japan).

### 4.3. Selenoprotein P Level Determination

For the measurement of full-length selenoprotein P concentrations in CSF and serum, we sandwiched the ELISA system with an AA3 method. Clone AA3 antibody recognizing the selenoprotein P C-terminal region was, therefore, used as a capture antibody. In addition, clone AH5 recognizing the N-terminal region of selenoprotein P was labeled with horseradish peroxidase (HRP) and used as a detection antibody. These antibodies were prepared as previously described [64], and human selenoprotein P was purified from human plasma, which was provided by the Japanese Red Cross Tohoku Block Blood Center (No. 25J0012).

Ninety-six-well microtiter plates were coated for 18 h at 4 °C with rat anti-human selenoprotein P monoclonal antibody AA3 (5 μg/mL) in 0.05 M sodium bicarbonate buffer, pH 9.6, filtered before use. The wells were washed four times with PBS containing 0.05% Tween 20 (200 µL) and incubated at 37 °C with PBS containing Block Ace (UK-B80, KAC Co., Kyoto, Japan) for 1 h after washing the wells four times, selenoprotein P standard or CSF or serum sample (diluted with PBS, containing 0.05% Tween 20 and 0.1% bovine serum albumin, PBS–Tween–BSA) was added to each well and incubated at 37 °C for 1 h after washing the wells four times, HRP-conjugated rat anti-human selenoprotein P monoclonal antibody AH5 (20 μg/mL) was added and incubated at 37 °C for 1 h. Finally, TMB Peroxidase Substrate (5120-0047, SeraCare, Life Science, Milford, MA, USA) was added to each well, and the protein–substrate reaction was allowed to proceed for 10 min. The reactions were stopped with the addition of 1 M sulfuric acid, and the absorbances were read at 450 nm in a SpectraMax iD5 (Molecular Devices, San Jose, CA, USA). We determined at least four points in each CSF or serum sample, and the average value was used for analysis. This assay was shown to be highly reproducible when identical samples were compared intra-assay or inter-assay, with the relative standard deviations within determinations under 5.0% and 10.0%, respectively. Overall, the laboratory personnel performed selenoprotein P determinations while blinded to patient diagnosis.

For all individuals diagnosed with AD and MCI subjects, we could also rely on CSF selenoprotein-P-bound selenium levels from a recent cohort study assessing whether the central nervous system selenium status could influence MCI progression to dementia [58]. In that study, inductively coupled plasma dynamic reaction cell mass spectrometry was used to determine the amount of selenium bound to selenoprotein P in CSF.

### 4.4. Statistical Analysis

We used spline regression analysis to assess the correlation of full-length selenoprotein P concentrations in CSF with its serum levels and with biomarkers of neurodegeneration (t-tau, p-tau181) and amyloidosis (Aβ1-42) in CSF, as well as selenoprotein-P-bound selenium. In more detail, we used a restricted cubic spline model with three knots at fixed percentiles (10th, 50th and 90th), adjusting for age at sample collection, sex and education. We also used linear regression analysis to assess the linear trend of some associations, by computing its β coefficient and the related 95% confidence interval (CI). We computed the crude and adjusted odds ratio (OR) for AD, MCI and ALS, by increasing 10-unit selenoprotein P concentrations through crude and multivariable unconditional logistic regression adjusting for sex and age as continuous variables. We also modeled the relation between selenoprotein P and disease risk using the restricted cubic spline model with three knots at fixed percentiles (10th, 50th and 90th), adding sex and age in the multivariable model. We used Stata 17.0 SE statistical package (StataCorp, College Station, TX, USA, 2021) for all statistical analyses. 

## Figures and Tables

**Table 1:** Cerebrospinal fluid (CSF) and serum levels of selenoprotein P and CSF levels of selenoprotein-P-bound selenium, amyloid-beta and phosphorylated and total tau in relation to diagnosis.

	Group ^Ɨ^			
	AD (*n* = 30)	MCI (*n* = 54)	ALS (*n* = 50)	Controls (*n* = 30)
Selenoprotein P (ng/mL)				
CSF	26.3 (21.0–39.1)	28.7 (19.8–36.1)	18.7 (13.1–26.1)	22.1 (14.9–29.9)
Serum	6012 (5595–6319)	6952 (6191–7686)	6623 (5817–7136)	5615 (4683–6235)
Selenoprotein-P-bound selenium in CSF (ng/mL)	1.4 (0.8–1.9)	1.6 (1.1–2.0)	-	-
Neurodegeneration biomarkers (ng/L)				
Amyloid-beta_1–42_	447 (385–494)	600 (441–806)	-	-
Phosphorylated tau	94 (73–105)	68 (49–88)	-	-
Total tau	568 (433–782)	337 (219–614)	-	-

^Ɨ^ Median (interquartile range).

**Table 2:** Odds ratio (OR) and 95% confidence interval (CI) of Alzheimer’s disease (AD), mild cognitive impairment (MCI) and amyotrophic lateral sclerosis (ALS) versus controls for 10-unit continuous increase in selenoprotein P levels.

	Univariate Model			Adjusted ^Ɨ^ Model		
	AD	MCI	ALS	AD	MCI	ALS
Selenoprotein P (ng/mL)						
CSF	1.33 (0.90–1.97)	1.28 (0.86–1.90)	0.72 (0.48–1.08)	1.26 (0.83–1.91)	1.12 (0.69–1.79)	0.63 (0.40–1.00)
Serum	1.01 (1.00–1.01)	1.01 (1.01–1.02)	1.01 (1.00–1.02)	1.01 (1.00–1.01)	1.02 (1.01–1.03)	1.01 (1.00–1.02)

^Ɨ^ Adjusted for age and sex.

## Data Availability

The data presented in this study may be available on reasonable request from the corresponding author. The data are not publicly available due to privacy and legal restrictions.

## Supplementary Materials

The supporting information can be downloaded at: https://www.mdpi.com/article/10.3390/ijms23179865/s1.
