# Supplementary material for: Selenoprotein P Concentrations in the Cerebrospinal Fluid and Serum of Individuals Affected by Amyotrophic Lateral Sclerosis, Mild Cognitive Impairment and Alzheimer’s Dementia
Source: Int J Mol Sci. 2022 Aug 30;23(17):9865. doi: 10.3390/ijms23179865 (PMC9456314; doi:10.3390/ijms23179865)
Supplement: Supplementary file 1 [file ijms-23-09865-s001.zip › ijms-1860099-supplementary.pdf]

International Journal of *Molecular Sciences*

MDPI

Supplemental Material

Table S1. Characteristics of the study population along with median and interquartile range (IQR) levels of selenoprotein P levels in cerebrospinal fluid (CSF) and serum according to sex, age, and education categories.

Characteristics	N	%	CSF (n=164) Selenoprotein P 50th (IQR)	Serum (n=153) Selenoprotein P 50th (IQR)
All participants	164	100	22.8 (15.3-32.9)	6364.6 (5670.3-6998.0)
Sex				
Men	87	53.0	26.1 (15.3-35.8)	6533.5 (5804.9-7126.3)
Women	77	47.0	22.1 (15.3-31.7)	6182.2 (5652.3-6952.1)
Age (years)				
< 65	84	51.2	22.8 (14.3-32.3)	6190.0 (5709.6-6989.2)
≥ 65	80	48.8	23.2 (16.9-33.3)	6528.2 (5595.6-7001.3)
Education (years) ǂ				
< 8	39	32.2	28.3 (20.6-33.0)	6630.3 (5766.6-7339.4)
≥ 8 and ≤ 12	40	33.1	23.1 (13.7-37.0)	6364.6 (5800.5-7136.4)
≥ 13	42	34.7	22.6 (14.3-33.8)	6558.8 (5703.4-6984.6)

ǂ Education information missing for 43 subjects (1 Alzheimer's dementia, 20 healthy controls, 22 amyotrophic lateral sclerosis).
